# Supplementary material for: The chronic effects of change of direction during repeated-sprint training on jumping, sprinting, and change-of-direction abilities in players: a systematic review and meta-analysis
Source: PeerJ. 2025 May 20;13:e19416. doi: 10.7717/peerj.19416 (PMC12101437; doi:10.7717/peerj.19416)

Annex 1: Excluded articles and reasons after reading the full text

| **Number** | **Name** | **Study** | **Reasons for exclusion** |
| --- | --- | --- | --- |
| 1 | Dal Pupo (2013) | Physiological and neuromuscular responses in the shuttle and straight line-repeated sprint running | Correlation study |
| 2 | Padulo (2015) | Repeated sprint ability in young basketball players: one vs. two changes of direction (Part 1) | Correlation study |
| 3 | Padulo (2016) | Repeated Sprint Ability in Young Basketball Players: Multi-direction vs. One-Change of Direction (Part 1) | Correlation study |
| 4 | Brini (2021) | Variation in Lower Limb Power and Three Point Shot Performance Following Repeated Sprints: One vs. Five Changes of Direction in Male Basketball Players | Correlation study |
| 5 | Born (2016) | Multi-Directional Sprint Training Improves Change-Of-Direction Speed and Reactive Agility in Young Highly Trained Soccer Players | Self-control study |
| 6 | Taylor (2016) | Two Weeks of Repeated-Sprint Training in Soccer: To Turn or Not to Turn? | Self-control study |
| 7 | Ozgunen (2021) | Effect of repeated sprint training on isokinetic strength parameters in youth soccer players | Self-control study |
| 8 | Chen (2019) | 多方向重复冲刺跑训练对少年儿童YOYO测试成绩及认知能力的影响研究  Study on the Impact of Multi-directional Repeated Sprint Training on YOYO Test Scores and Cognitive Abilities in Children and Adolescents | Inconsistent subjects |
| 9 | Shi (2021) | 多方向重复冲刺跑练习对高年级小学生身体素质影响的实验研究  Experimental Study on the Impact of Multi-directional Repeated Sprint Exercises on the Physical Fitness of Higher-grade Primary School Students | Inconsistent subjects |
| 10 | Morais (2025) | Effects of On-Court Tennis Training Combined with HIIT versus RST on Aerobic Capacity, Speed, Agility, Jumping Ability, and Internal Loads in Young Tennis Players | RST+On-ourt tennis training (OTT) |
| 11 | Brini (2023) | Neuromuscular and balance adaptations following basketball-specific training programs based on combined drop jump and multidirectional repeated sprint versus multidirectional plyometric training | RST+DJ |
| 12 | Brini (2022) | Impact of combined versus single-mode training programs based on drop jump and specific multidirectional repeated sprint on bio-motor ability adaptations: a parallel study design in professional basketball players | RST+DJ |
| 13 | Kilit (2025) | Effects of 2 Combined Training Protocols on the Aerobic and Anaerobic Fitness, Technical Skills, and Psychophysiological Responses in Young Soccer Players | RST+ small-sided soccer games (SSGs) |
| 14 | Tian (2020) | 折返重复冲刺训练对青少年篮球运动员运动能力和下肢肌肉爆发力的影响  The Effects of Repeated Sprint Training with Change of Direction on Physical Performance and Lower Limb Muscle Power in Adolescent Basketball Players | RST with no COD |

Annex 2: Effect sizes and prediction intervals for each subgroup

CMJ: RS-OCOD


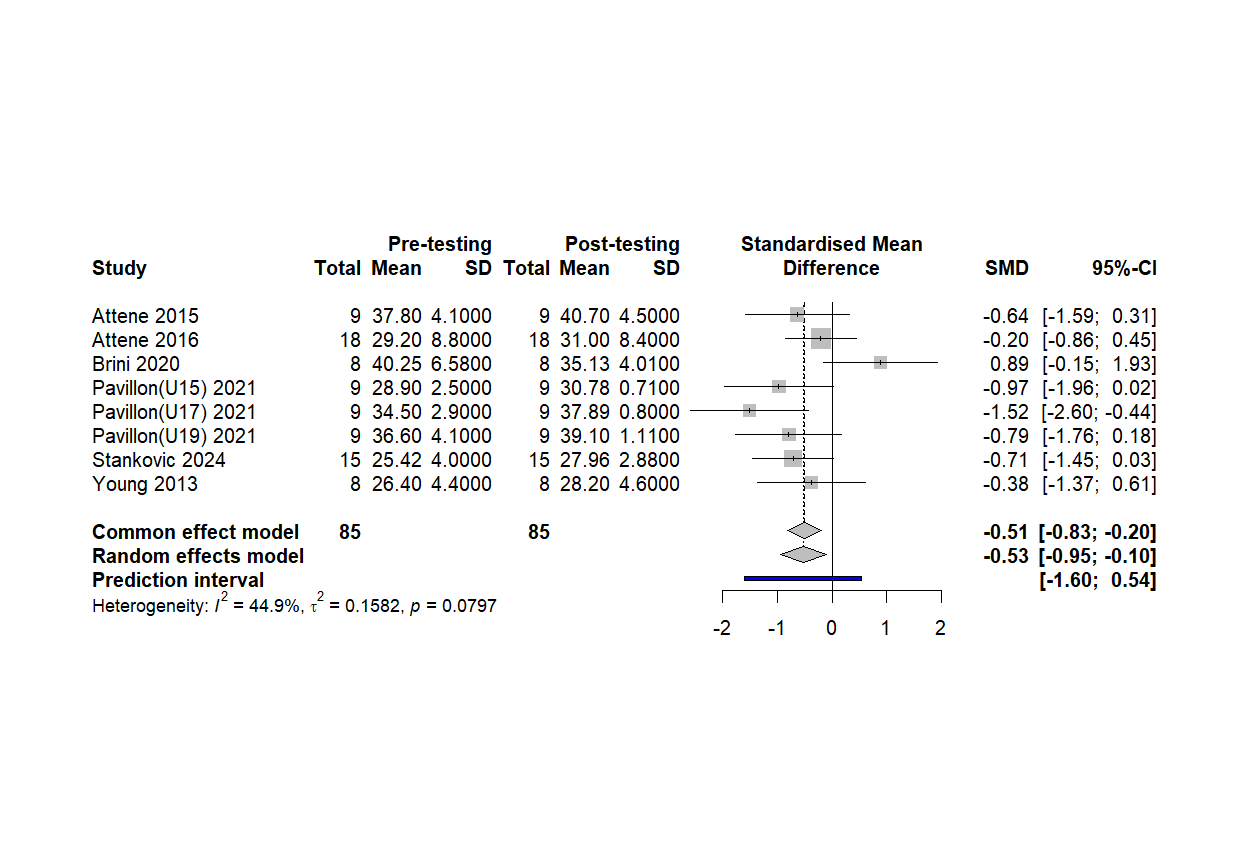


CMJ: RS-MCOD


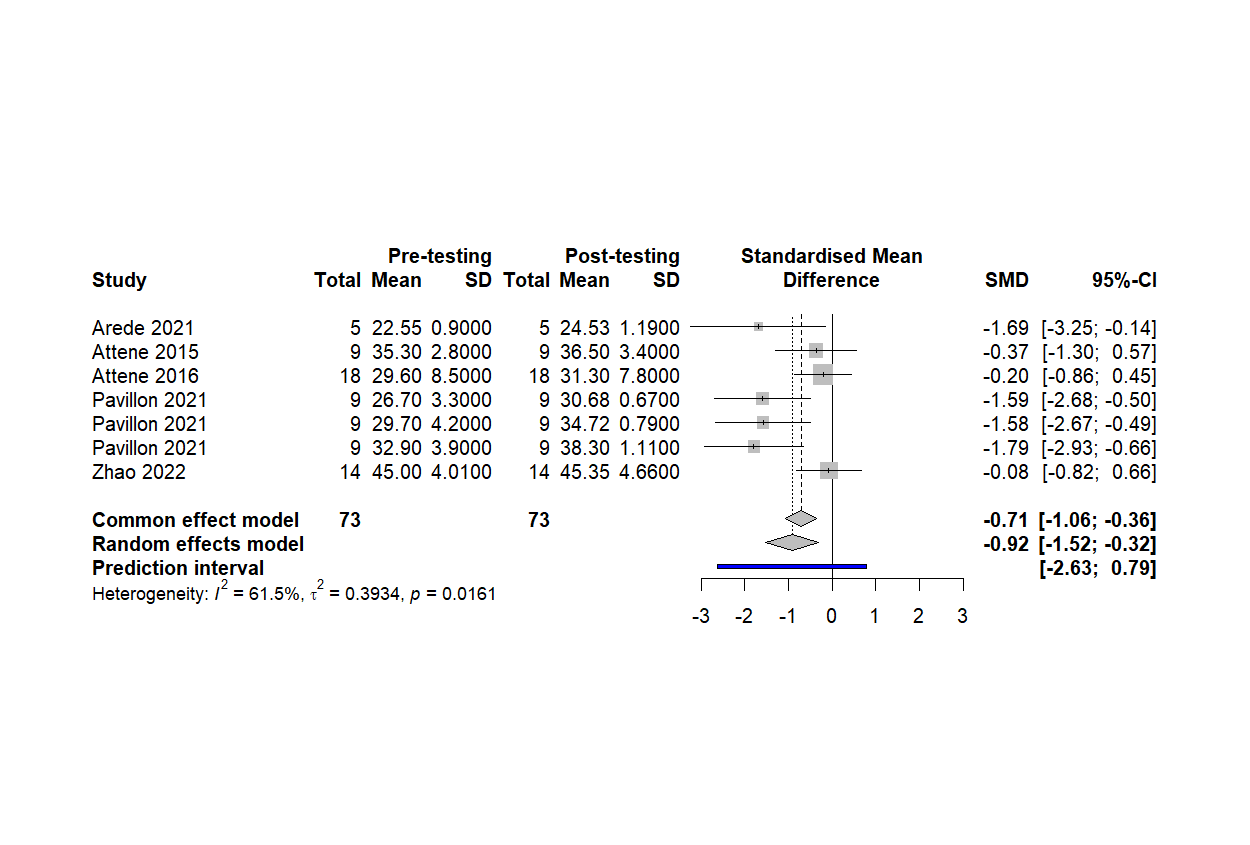
SJ: RS-OCOD


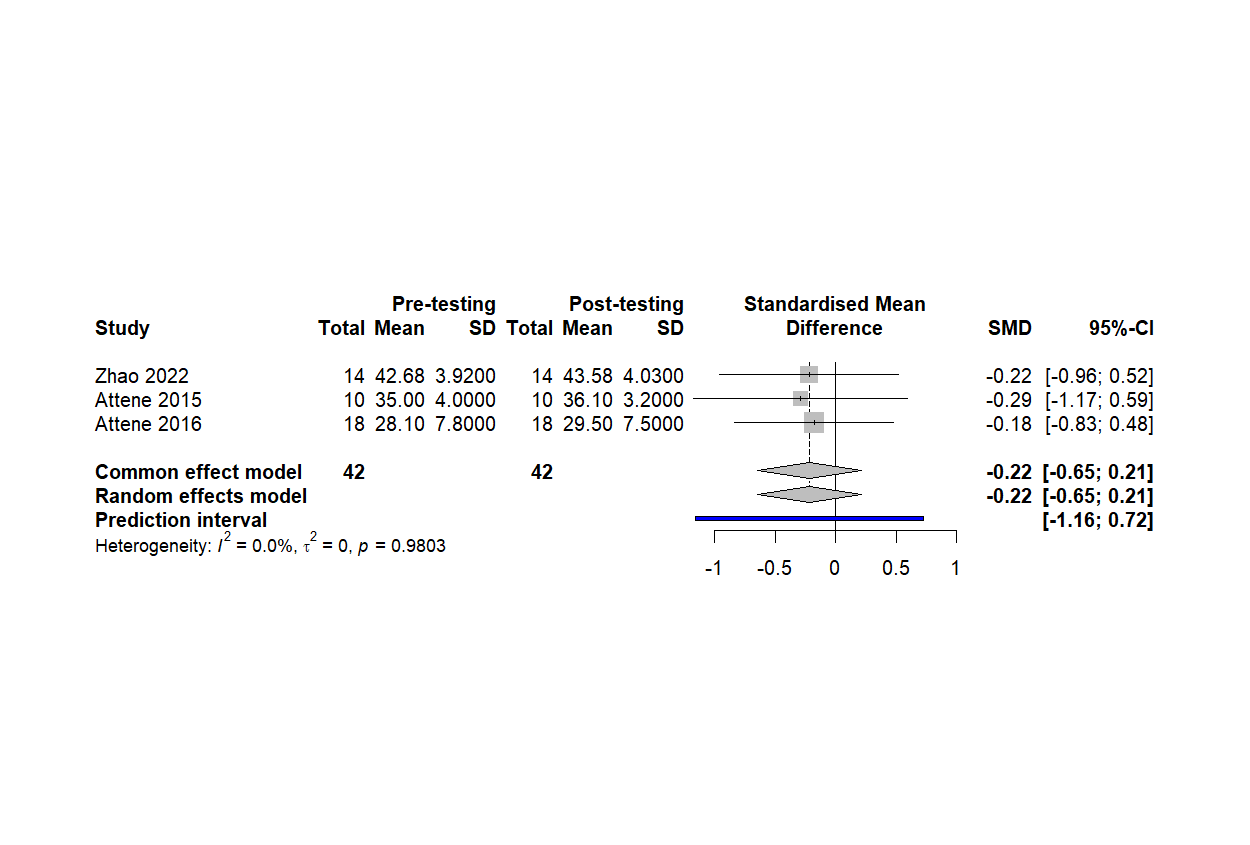


SJ: RS-MCOD


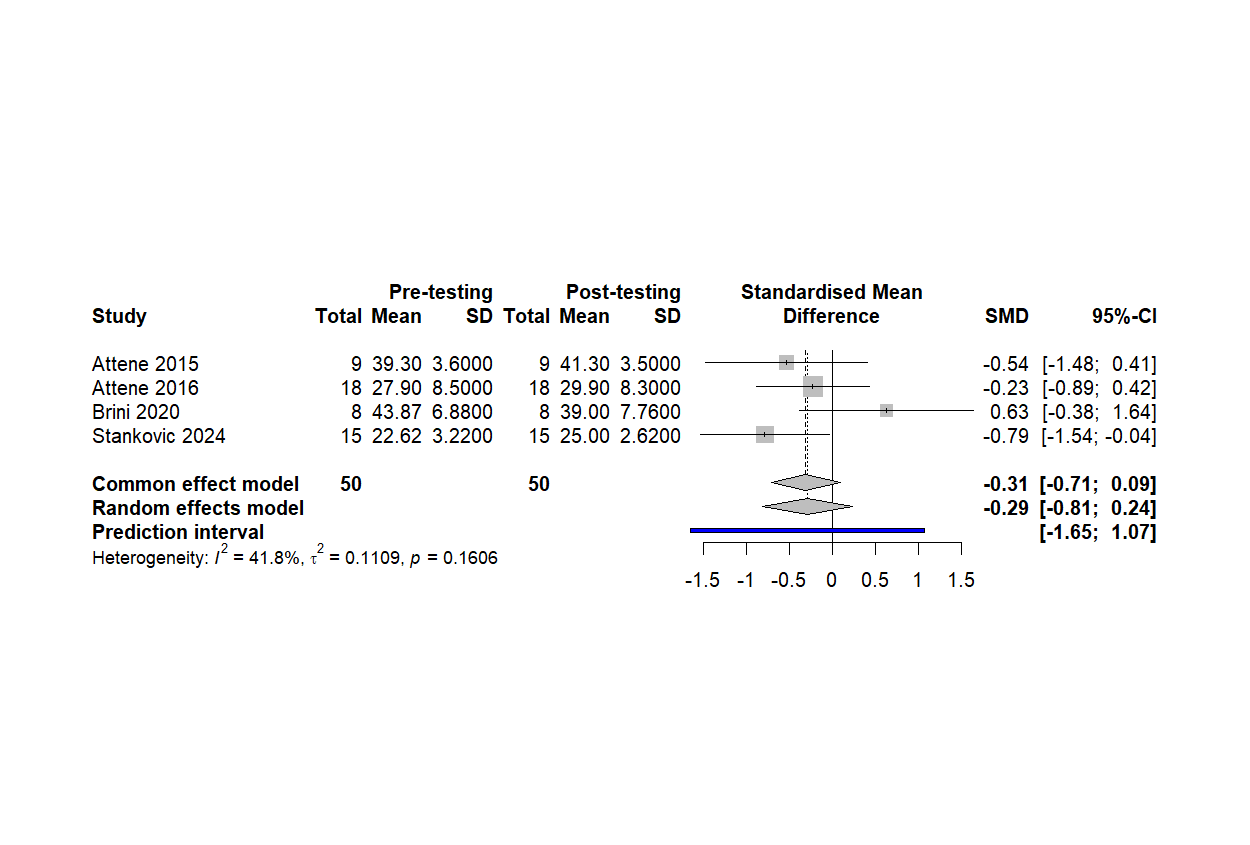


5m: RS-OCOD


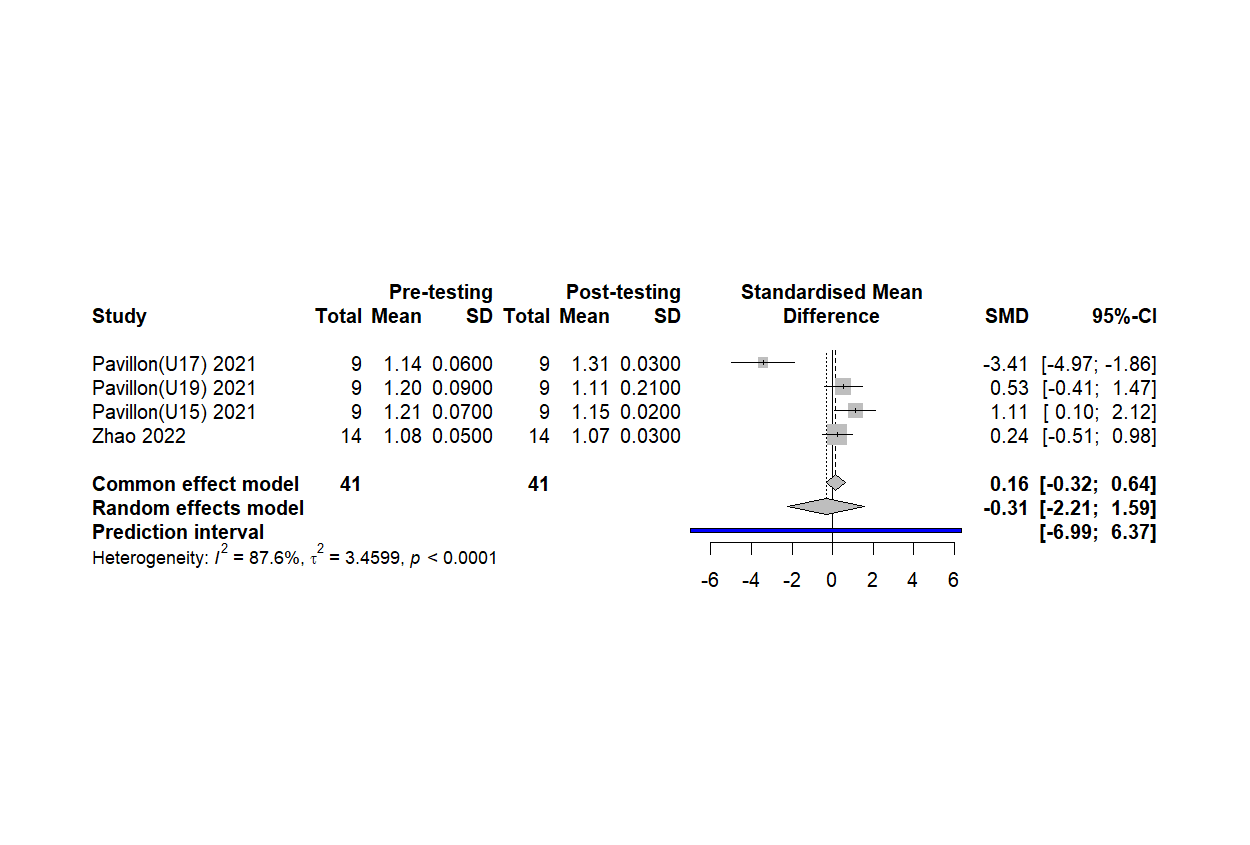


5m: RS-MCOD


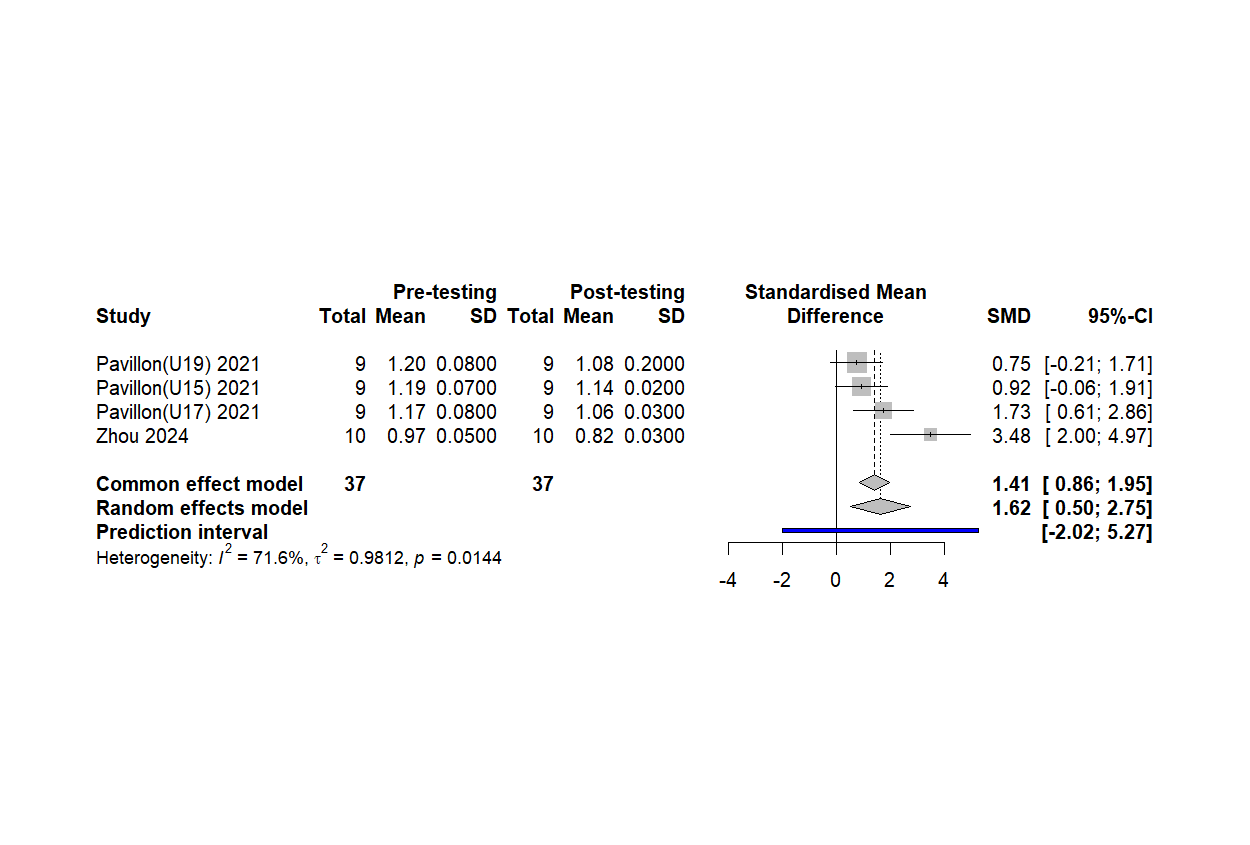


10m: RS-OCOD


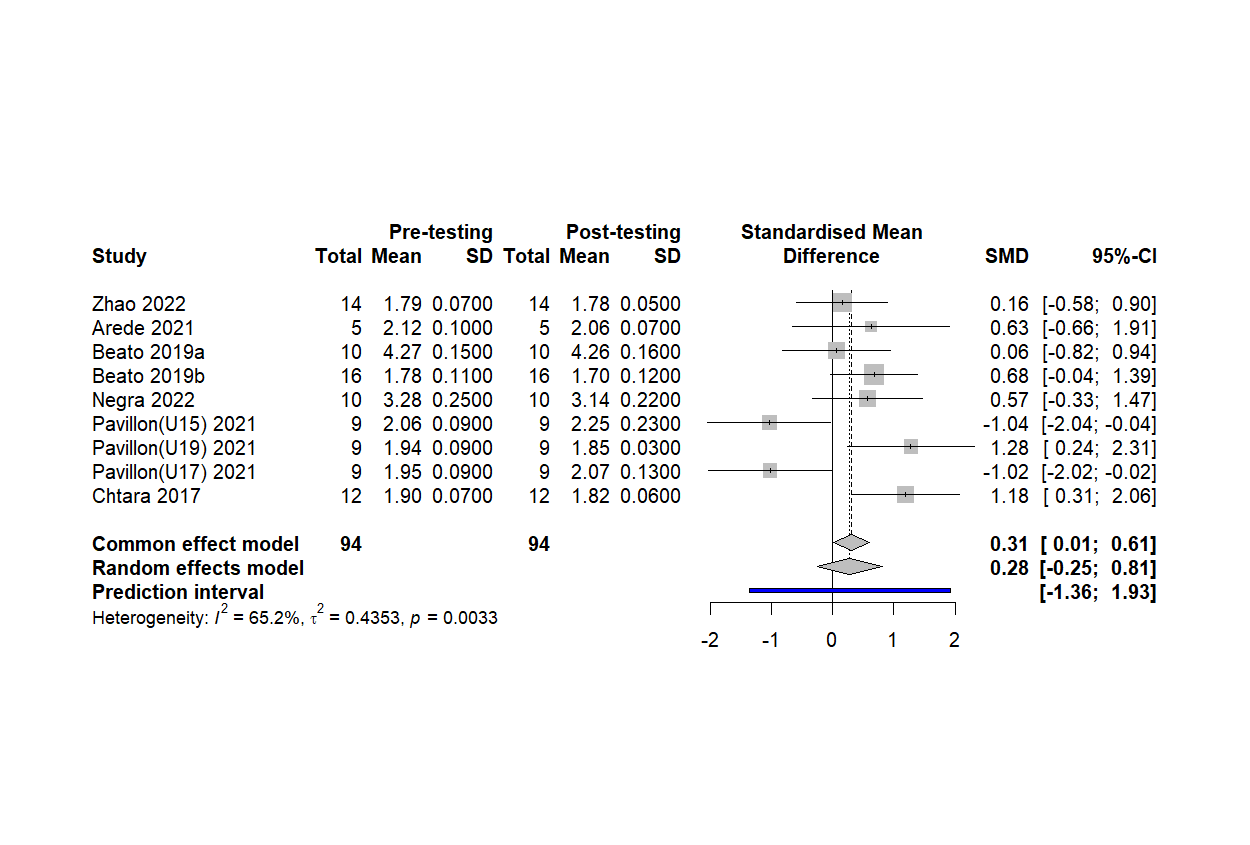


10m: RS-MCOD


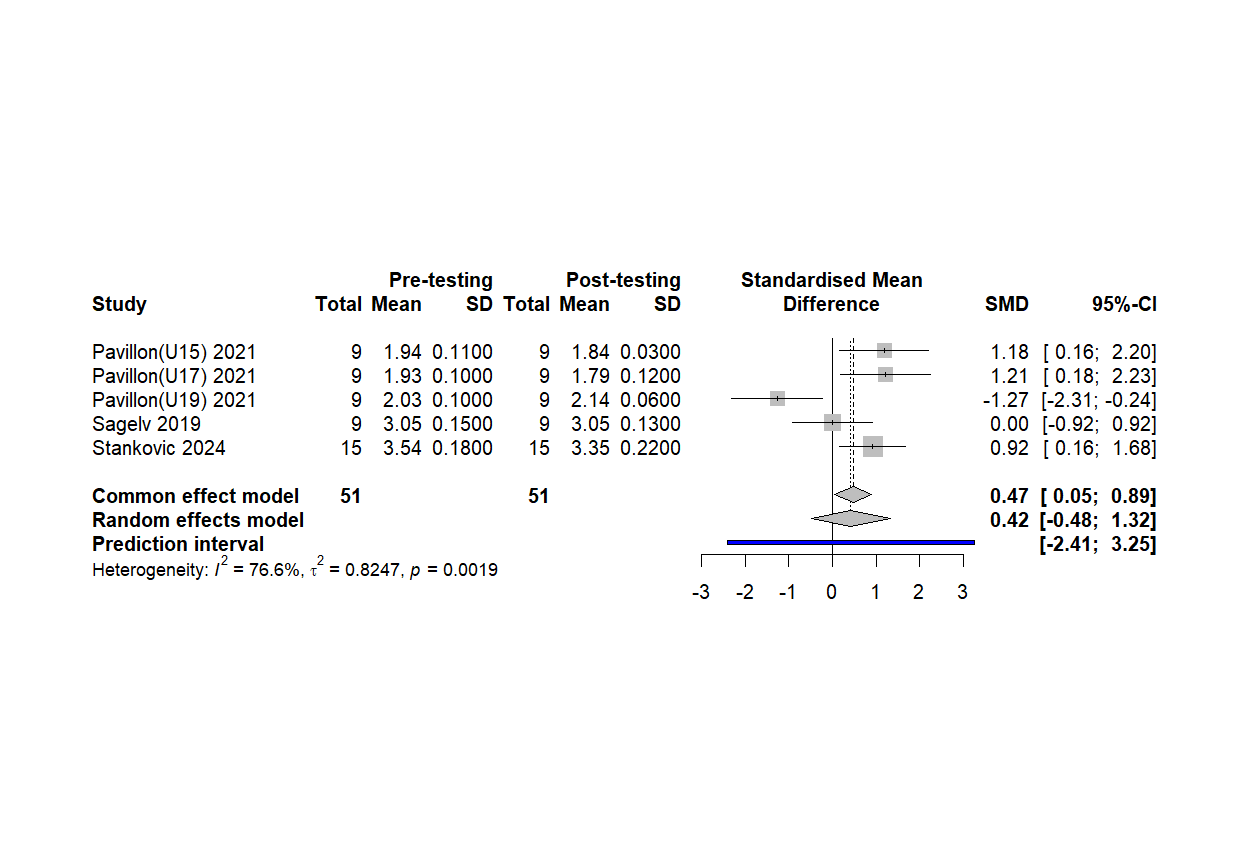


20-40m: RS-OCOD


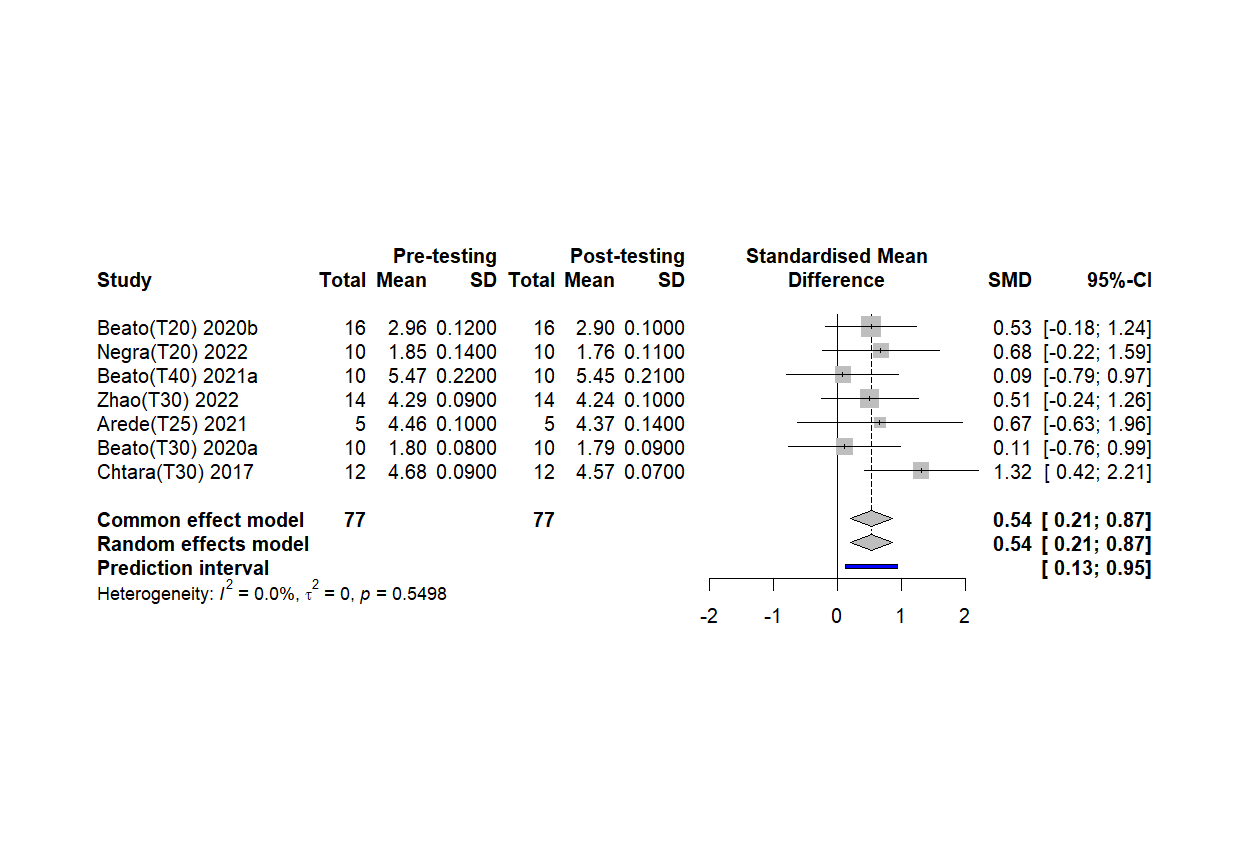


20-40m: RS-MCOD


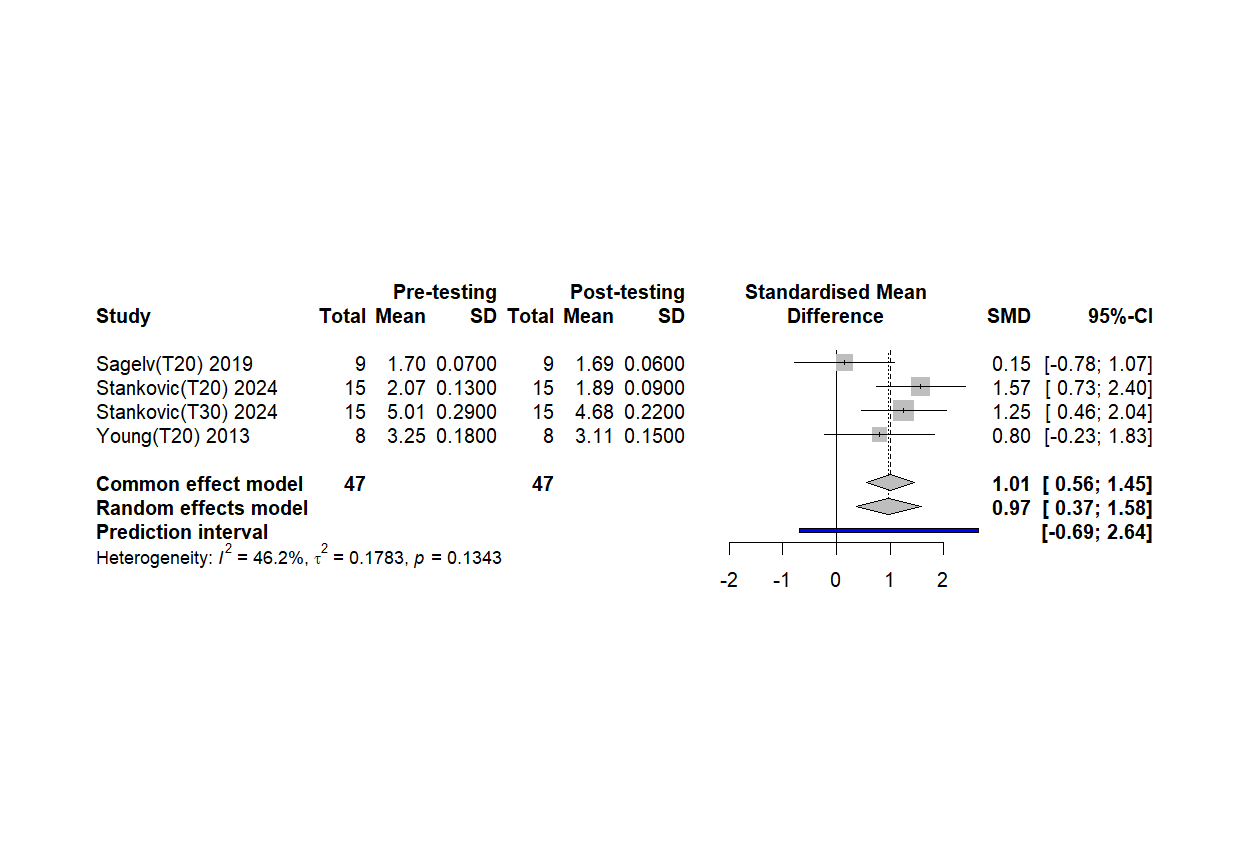


COD: RS-OCOD


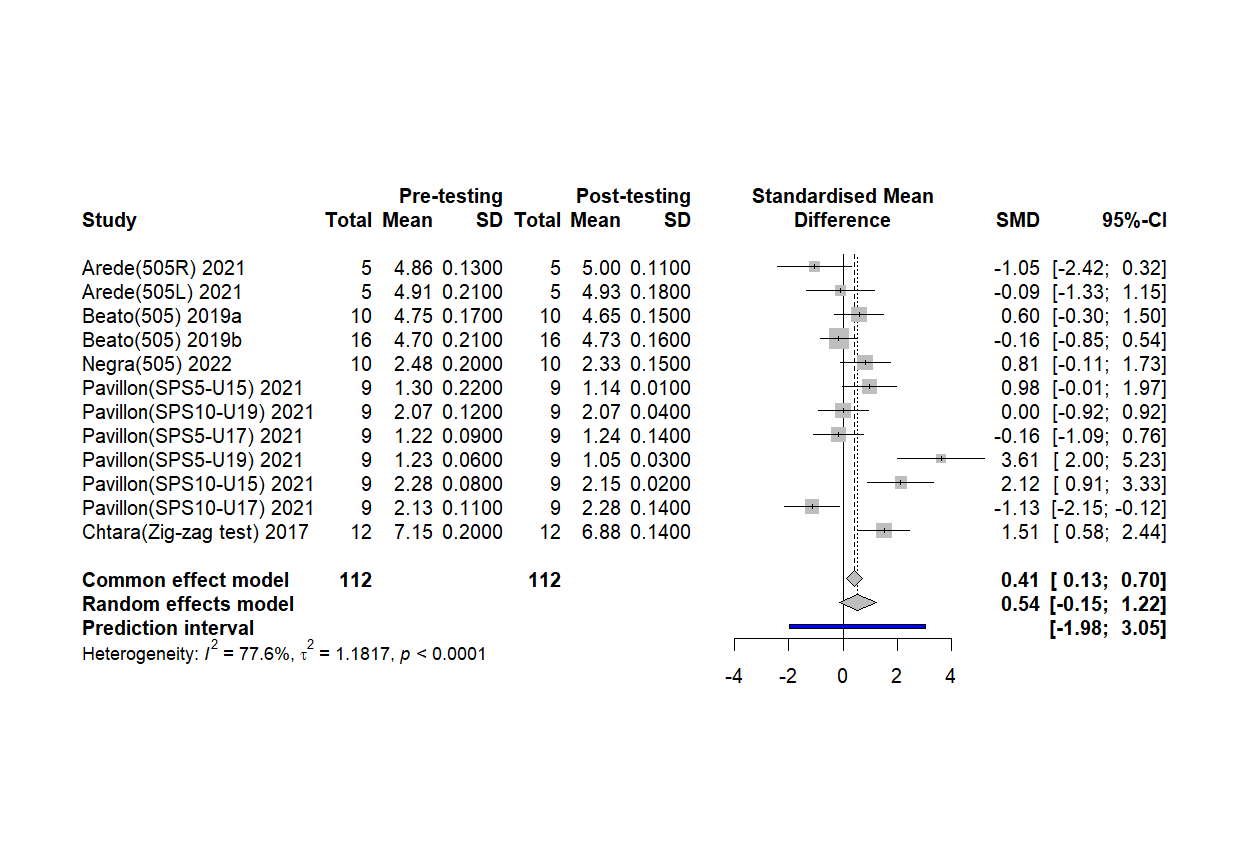


COD: RS-MCOD


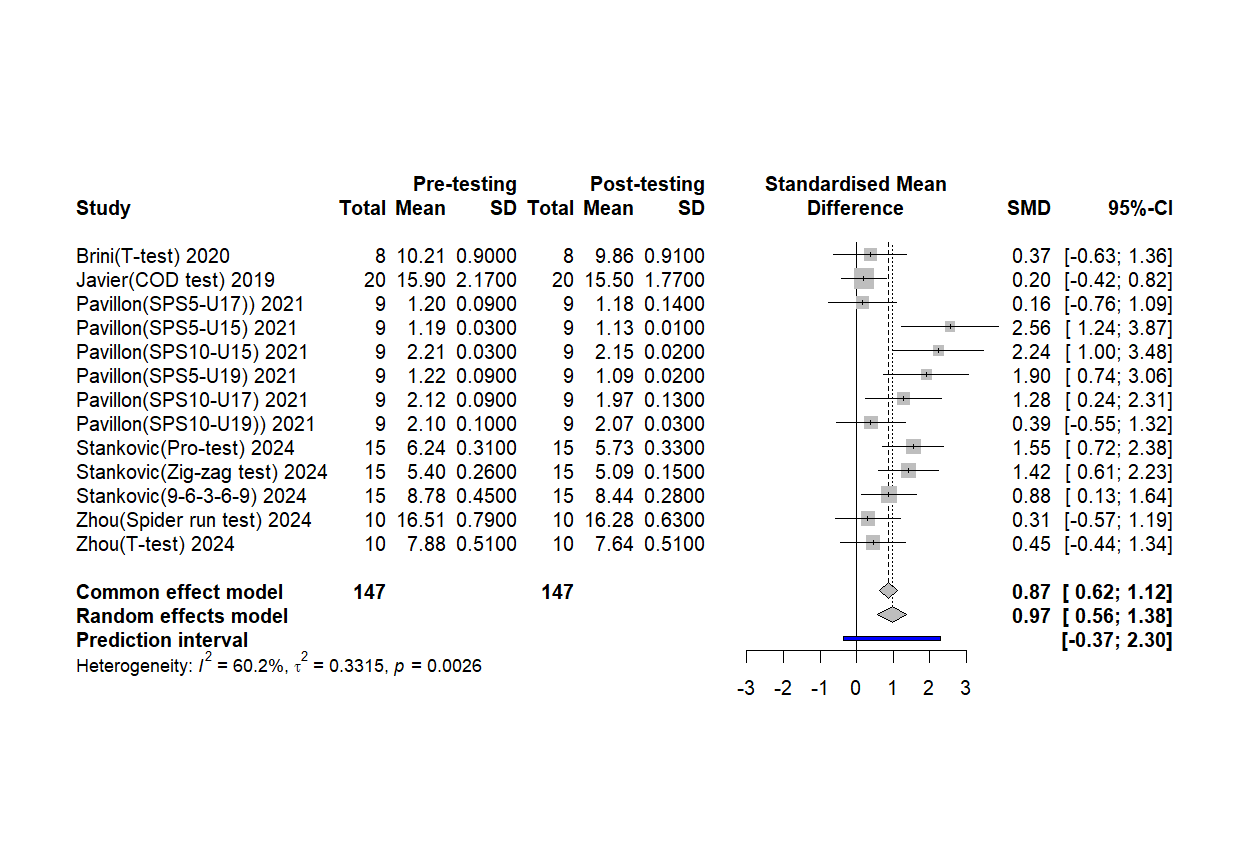

Supplement: Supplemental Information 1 [file peerj-13-19416-s001.docx]
